# The leukocyte glucose index: a novel inflammatory-glucose biomarker for prevalent diabetic retinopathy and its supplementary predictive capacity in individuals with type 2 diabetes mellitus

**DOI:** 10.3389/fendo.2026.1959346

**Published:** 2026-09-07

**Authors:** Shimeng Huang, Li Wang, Cong Zhang, Ruoxuan Zheng, Xiaotong Sun, Jingjing Li, Siyu Dong, Rui Hou, Shuang Wu, Xinyu Zhang, Tianpeng Zhang, Chengye Xu, Hongyu Kuang

**Affiliations:** Department of Endocrinology, The First Affiliated Hospital of Harbin Medical University, Harbin, Heilongjiang, China

**Keywords:** diabetic retinopathy, LASSO, leukocyte glucose index, nomogram, type 2 diabetes mellitus

## Abstract

**Background:**

The Leukocyte Glucose Index (LGI) reflects systemic inflammation and glycemic burden and has shown promising predictive value in cardiovascular and metabolic diseases. However, its association with prevalent diabetic retinopathy (DR) remains unclear. We investigated the association and dose–response relationship between LGI and prevalent DR in patients with T2DM and its incremental value beyond traditional clinical factors.

**Methods:**

1, 727 hospitalized patients with T2DM, including 661 with DR and 1, 066 without DR. Multivariable logistic regression, subgroup, trend, and restricted cubic spline analyses were performed to assess the association between LGI and prevalent DR. Variables selected by LASSO regression and multivariable logistic regression were used to develop a nomogram. Model performance was evaluated by discrimination, calibration, bootstrap internal validation, reclassification metrics, and decision curve analysis (DCA).

**Results:**

Higher LGI was independently associated with prevalent DR (adjusted OR = 1.340, 95% CI: 1.056–1.701, *P* = 0.016). Similar results were observed per standard deviation increase (adjusted OR = 1.166, 95% CI: 1.029–1.320) and in the highest versus lowest tertile (adjusted OR = 1.378, 95% CI: 1.028–1.848). A positive linear association was identified without evidence of nonlinearity. The nomogram achieved an AUC of 0.727 (95% CI: 0.703–0.752), with an optimism-corrected AUC of 0.719. Although the improvement in AUC after adding LGI was not statistically significant (0.727 vs. 0.725, *P* = 0.198), NRI and IDI indicated improved discrimination, and DCA showed greater net clinical benefit across a range of threshold probabilities.

**Conclusions:**

Elevated LGI was independently associated with prevalent DR and showed a positive linear dose–response relationship. LGI may provide incremental discriminative value beyond traditional clinical factors and serve as a simple laboratory marker for identifying prevalent DR. Further external validation and prospective studies are warranted.

## 1 Introduction

According to the International Diabetes Federation, more than 500 million adults worldwide were living with diabetes in 2024, and this number is projected to increase to approximately 900 million by 2050, imposing an increasing burden on healthcare systems globally (1). Diabetes is associated with a wide range of chronic complications, among which diabetic retinopathy (DR) is a leading cause of visual impairment and blindness among working-age adults. Approximately one-third of individuals with diabetes are affected by DR to varying degrees, and severe cases may result in permanent vision loss (2, 3). Therefore, timely identification of DR and appropriate intervention are essential for reducing the risk of severe visual impairment.

However, DR is often asymptomatic in its early stages, and its diagnosis and staging rely on fundus imaging and interpretation by experienced ophthalmologists. With the growing number of patients with DR, the availability of trained retinal specialists and imaging equipment remains limited, particularly in resource-constrained settings. Consequently, access to conventional fundus screening may be restricted, potentially delaying the identification of DR and increasing the burden on both patients and healthcare systems (4–7). Therefore, exploring simple and readily accessible indicators that can assist in DR risk stratification may help optimize screening strategies.

The development and progression of DR involve multiple pathophysiological mechanisms, including chronic hyperglycemia, accumulation of advanced glycation end products, oxidative stress, and low-grade chronic inflammation (3, 8). Although glycated hemoglobin (HbA1c), blood glucose levels, and diabetes duration have been established as important factors associated with DR (9–13), these indicators mainly reflect a single dimension of glycemic status or disease exposure and may not fully capture the combined effects of inflammation and metabolic abnormalities.

The Leukocyte Glucose Index (LGI), calculated using peripheral leukocyte count and fasting plasma glucose (FPG), is a simple, inexpensive, and readily available composite indicator that may reflect both systemic inflammatory status and glycemic burden (14, 15). In recent years, LGI has attracted increasing attention in cardiovascular diseases, metabolic disorders, and critically ill conditions (16–19). Previous studies have shown that inflammatory markers, such as the neutrophil-to-lymphocyte ratio (NLR) and monocyte-to-lymphocyte ratio (MLR), as well as metabolic indicators including FPG and the cholesterol–high-density lipoprotein–glucose (CHG) index, are associated with DR (9, 20–23). Compared with individual inflammatory or metabolic markers, LGI integrates information on both inflammation and glucose metabolism and therefore has a sound biological rationale. However, the independent association between LGI and DR, as well as its incremental discriminative value beyond traditional clinical factors, has not yet been investigated.

Therefore, this study aimed to investigate the association and dose–response relationship between LGI and prevalent DR in patients with type 2 diabetes mellitus (T2DM), evaluate the incremental value of LGI beyond traditional clinical factors, and further develop a nomogram incorporating LGI to provide additional information for individualized risk stratification and the identification of prevalent DR.

## 2 Materials and methods

### 2.1 Study population

This single-center cross-sectional study was conducted at the First Affiliated Hospital of Harbin Medical University. The study was performed in accordance with the ethical principles of the Declaration of Helsinki and was approved by the Ethics Committee of the First Affiliated Hospital of Harbin Medical University. Clinical data were consecutively collected from hospitalized patients with T2DM between July 2024 and July 2025.

The inclusion criteria were as follows: (1) age ≥18 years; (2) diagnosis of T2DM; and (3) complete clinical and laboratory data.

The exclusion criteria were: (1) other types of diabetes; (2) active or chronic inflammatory diseases, autoimmune diseases, or acute or chronic hepatic or renal insufficiency; (3) a history of malignancy; (4) severe ocular diseases, including glaucoma, retinal vein occlusion, and retinal detachment; and (5) missing key clinical or laboratory data.

### 2.2 Definition of DR

All participants were evaluated by experienced ophthalmologists based on ophthalmic examinations according to the International Clinical Diabetic Retinopathy Disease Severity Scale. DR was diagnosed when any characteristic retinal lesion defined by the Early Treatment Diabetic Retinopathy Study (ETDRS) criteria was present.

### 2.3 Clinical and laboratory data

All patients were hospitalized between July 2024 and July 2025. Demographic characteristics, medical history, and laboratory data were obtained from the hospital electronic medical record system and outpatient follow-up records. Clinical variables included sex, age, diabetes duration, smoking status, alcohol consumption, body mass index (BMI), history of hypertension, history of cardiovascular disease (CVD), insulin use, and Oral antidiabetic drug. BMI was calculated as weight (kg) divided by height squared (m²).

All laboratory measurements were performed using fasting venous blood samples collected before 08:00 on the morning after hospital admission. HbA1c was measured by high-performance liquid chromatography. Alanine aminotransferase (ALT), aspartate aminotransferase (AST), albumin (ALB), FPG, total cholesterol (TC), triglycerides (TG), low-density lipoprotein cholesterol (LDL-C), high-density lipoprotein cholesterol (HDL-C), Urea, uric acid (UA), and serum creatinine (Cr) were determined using standard enzymatic methods. Leukocyte count was measured using an automated hematology analyzer (Sysmex XT-2000i, Sysmex, Japan). The LGI was calculated as: LGI = WBC (×10^9^/L) × FPG (mg/dL) × 0.001 (15, 18).

### 2.4 Statistical analysis

All statistical analyses were performed using SPSS version 27.0 (IBM Corp., Armonk, NY, USA), EmpowerStats version 2.0, Python version 3.8.13, and R version 4.3.3. Continuous variables were tested for normality. Normally distributed variables are presented as the mean ± standard deviation (SD) and were compared using the independent-samples *t* test. Non-normally distributed variables are presented as the median (interquartile range [IQR]) and were compared using the Mann–Whitney *U* test. Categorical variables are expressed as frequencies (percentages) and were compared using the χ² test.

Least absolute shrinkage and selection operator (LASSO) regression was used for variable selection. By imposing a penalty on the regression coefficients, LASSO shrinks some coefficients toward zero and excludes variables with coefficients shrunk to zero, thereby selecting a relatively parsimonious set of candidate variables and reducing the potential for overfitting. Ten-fold cross-validation was used to determine the penalty parameter λ, and the λ1se criterion was applied to identify a relatively parsimonious and stable set of variables for subsequent multivariable modeling and nomogram construction (24). Spearman correlation and partial correlation analyses were performed to evaluate the correlations between LGI and clinical variables related to DR. Age, sex, and BMI were prespecified as important clinical confounders and were adjusted for in all multivariable regression analyses regardless of whether they were retained by LASSO.

Multivariable logistic regression was performed to evaluate the association between LGI and prevalent DR. Three progressively adjusted models were constructed: Model 1 was unadjusted; Model 2 was adjusted for age, sex, BMI, and diabetes duration; and Model 3 was a clinically prespecified fully adjusted model that further included insulin use, hypertension, ALT, AST, Urea, Cr, TC, LDL-C, ALB, and HbA1c based on Model 2. Restricted cubic spline (RCS) analysis was performed to examine the dose–response relationship and potential nonlinear association between LGI and prevalent DR. Subgroup analyses and interaction tests were conducted to evaluate whether clinical characteristics modified the association between LGI and prevalent DR.

Model discrimination was assessed using receiver operating characteristic (ROC) curves and the area under the curve (AUC). Higher AUC values indicate better ability to discriminate between patients with and without prevalent DR. Generally, an AUC of 0.7–0.8 is considered acceptable discrimination, 0.8–0.9 good discrimination, and >0.9 excellent discrimination (25). Internal validation was performed separately for the base model and the nomogram using 1, 000 bootstrap resamples to assess model stability and obtain optimism-corrected AUCs, thereby reducing potential optimism in the apparent model performance. Model calibration was assessed using calibration curves, the Hosmer–Lemeshow goodness-of-fit test, and the Brier score. Calibration curves were plotted to visually assess model calibration. Calibration refers to the agreement between the model-predicted probability of DR and the observed event rate. The x-axis represents the predicted probability, and the y-axis represents the observed event rate. The 45° reference line indicates perfect calibration, where the predicted probabilities exactly match the observed outcomes. A calibration curve closer to the 45° reference line indicates better model calibration (26). Model fit and calibration were evaluated using the Hosmer–Lemeshow goodness-of-fit test based on the chi-square distribution, with a P value >0.05 indicating no statistically significant evidence of poor calibration (25). The Brier score quantifies the mean squared error between predicted probabilities and observed outcomes and ranges from 0 to 1 (lower values indicate better accuracy) (27). We used decision curve analysis (DCA) to evaluate the clinical utility of the model. DCA assesses the model’s potential clinical utility by evaluating its net benefit across a range of threshold probabilities, with a higher net benefit indicating greater potential clinical utility (28). We further used the net reclassification improvement (NRI) and integrated discrimination improvement (IDI) to assess the incremental discriminative value of adding LGI to the base model. NRI and IDI quantify the improvement in discriminative performance of the new model compared with the original model. An NRI or IDI > 0 indicates an improvement with the new model, whereas an NRI or IDI < 0 indicates a decline in discriminative performance, and an NRI or IDI = 0 indicates no incremental improvement (28, 29). All statistical tests were two-sided, and *P* value <0.05 was considered statistically significant.

## 3 Results

### 3.1 Baseline characteristics of the study population

A total of 1, 727 patients with T2DM were included in this study, comprising 661 patients with DR and 1, 066 without DR. The baseline characteristics of the study population are presented in **Table 1**. Compared with the non-DR (NDR) group, patients with DR were significantly different in age, diabetes duration, BMI, history of hypertension, insulin use, and levels of TC, LDL-C, ALT, AST, ALB, Urea, Cr, FPG, HbA1c, WBC, and LGI (all *P* < 0.05).

**Table 1:** The baseline characteristics of the study population.

Variables	Non-diabetic retinopathy (N = 1066)	Diabetic retinopathy (N = 661)	P value
Age (years)	55.00 (46.00-63.00)	56.00 (50.00-64.00)	0.002**
diabetes duration (years)	5.00 (1.00-10.00)	10.00 (5.00-18.00)	<0.001***
BMI (kg/m²)	25.51 (23.43-27.97)	25.34 (23.32-27.34)	0.015*
sex, n (%)			0.591
Female	417 (39.1%)	250 (37.8%)	
Male	649 (60.9%)	411 (62.2%)	
Hypertension history, n (%)			0.001**
No	622 (58.3%)	334 (50.5%)	
Yes	444 (41.7%)	327 (49.5%)	
insulin use, n (%)			<0.001***
No	617 (57.9%)	202 (30.6%)	
Yes	449 (42.1%)	459 (69.4%)	
Oral antidiabetic drug, n (%)			0.983
No	264 (24.8%)	164 (24.8%)	
Yes	802 (75.2%)	497 (75.2%)	
Smoking history, n (%)			0.549
No	769 (72.1%)	468 (70.8%)	
Yes	297 (27.9%)	193 (29.2%)	
Alcohol history, n (%)			0.407
No	828 (77.7%)	502 (75.9%)	
Yes	238 (22.3%)	159 (24.1%)	
CVD, n (%)			0.211
No	868 (81.4%)	522 (79.0%)	
Yes	198 (18.6%)	139 (21.0%)	
TC (mmol/L)	4.76 (3.95-5.56)	4.85 (4.06-5.71)	0.008**
TG (mmol/L)	1.76 (1.20-2.78)	1.80 (1.22-2.89)	0.747
HDL-C (mmol/L)	1.10 (0.96-1.30)	1.10 (0.95-1.30)	0.948
LDL-C (mmol/L)	2.96 (2.25-3.63)	3.07 (2.41-3.77)	0.008**
ALT (U/L)	22.75 (16.40-36.30)	19.20 (14.20-27.80)	<0.001***
AST (U/L)	20.60 (16.40-28.10)	18.80 (15.40-23.90)	0.002**
ALB (g/L)	42.80 (40.50-45.40)	42.20 (39.60-44.50)	<0.001***
Urea (mmol/L)	5.46 (4.62-6.58)	5.86 (4.94-7.09)	<0.001***
Cr (μmol/L)	63.00 (53.30-74.70)	65.00 (53.80-78.10)	<0.001***
UA (μmol/L)	330.00 (273.30-397.13)	335.90 (275.40-411.00)	0.450
FPG (mmol/L)	8.05 (6.79-10.01)	8.55 (6.65-11.11)	<0.001***
FPG (mg/dL)	145.03 (122.37-180.30)	154.04 (119.81-200.16)	<0.001***
HbA1c (%)	8.20 (7.10-9.50)	8.80 (7.50-10.30)	<0.001***
HbA1c (mmol/mol)	66.12 (54.10-80.33)	72.68 (58.47-89.07)	<0.001***
WBC	6.16 (5.23-7.28)	6.34 (5.32-7.59)	0.007**
LGI	0.90 (0.71-1.20)	0.97 (0.72-1.39)	<0.001***

Data are expressed as mean ± standard deviation, percentage, or median (IQR); p-values were compared using independent t-tests, Mann-Whitney U tests, or chi-square tests. Significance was set at *P < 0.05, **P < 0.01, ***P < 0.001. DR, diabetic retinopathy; BMI, body mass index; SBP, systolic blood pressure; DBP, diastolic blood pressure; CVD, cardiovascular disease; TC, Total Cholesterol; TG, Triglyceride; HDL-C, high-density lipoprotein cholesterol; LDL-C, low-density lipoprotein cholesterol; ALT, Alanine Aminotransferase; AST, Aspartate Aminotransferase; ALB, Albumin; Cr, Creatinine; FPG, Fasting Plasma Glucose; HbA1c, glycated hemoglobin; LGI, Leukocyte Glucose Index.

### 3.2 Variable selection using LASSO regression

**Figure 1:**
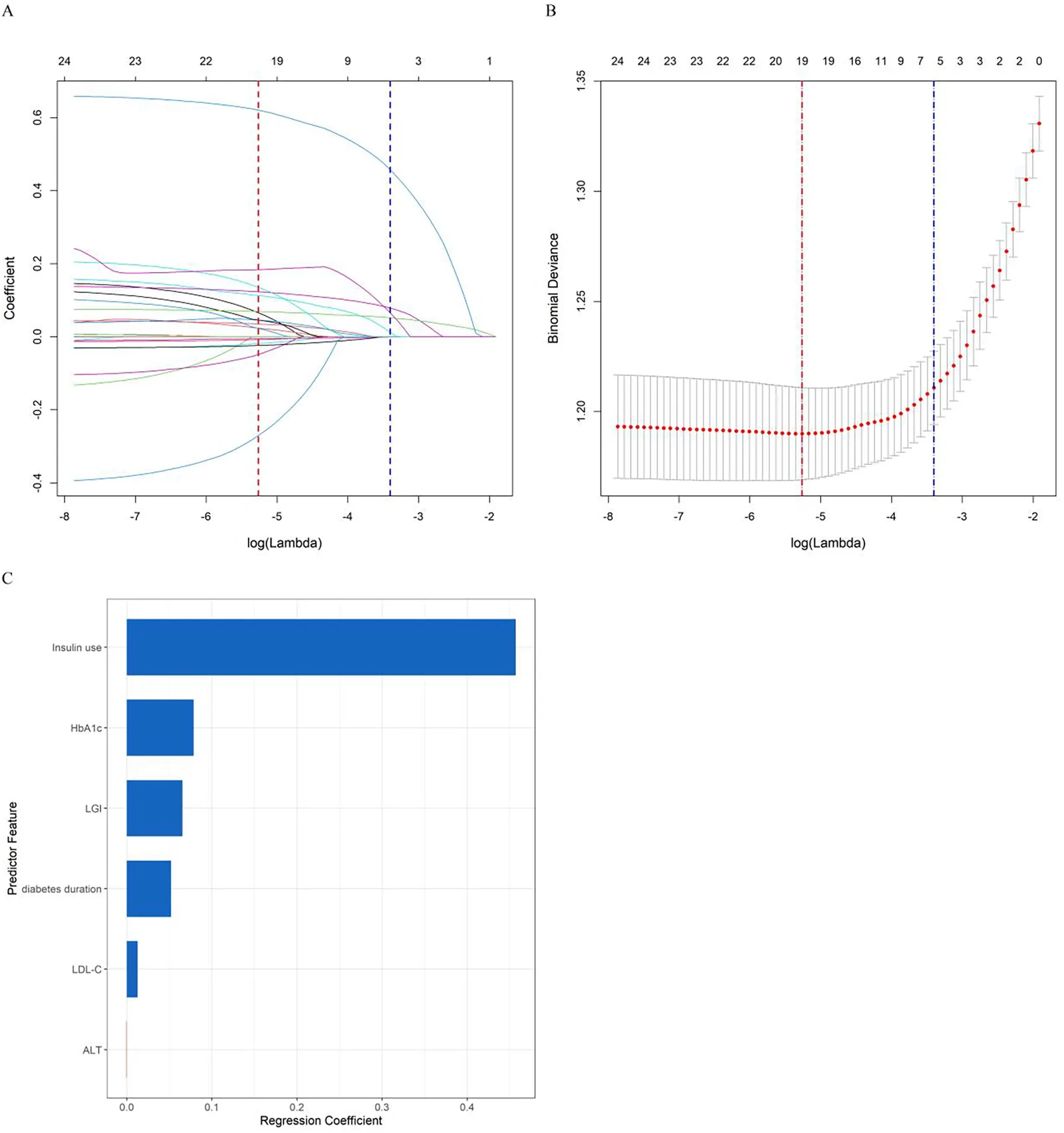
Variable selection using LASSO regression. **(A)** Coefficient profiles of the 24 candidate variables across different values of the penalty parameter λ; as λ increases, more coefficients are shrunk toward zero. **(B)** 10-fold cross-validation was used to select the penalty parameter λ; the vertical dashed lines represent λ.min (red) and λ.1-SE (blue). The λ.1-SE criterion was applied to select the largest λ within one standard error of the minimum cross-validated error, thereby yielding a relatively parsimonious set of variables. **(C)** The variables selected by the LASSO model and their regression coefficients at the λ.1-SE criterion. The numbers at the top of panels **(A, B)** indicate the number of nonzero coefficients at the corresponding λ values. Abbreviations are listed in **Table 1**.

To identify key variables associated with DR, LASSO regression was applied to 24 clinical and biochemical variables, and the optimal penalty parameter (λ) was determined using 10-fold cross-validation. According to the λ1se criterion, six variables with non-zero coefficients were selected, including insulin use, HbA1c, LGI, diabetes duration, LDL-C, and ALT (**Figure 1**). These variables were subsequently incorporated into the nomogram.

### 3.3 Association and dose–response relationship between LGI and prevalent DR

**Figure 2:**
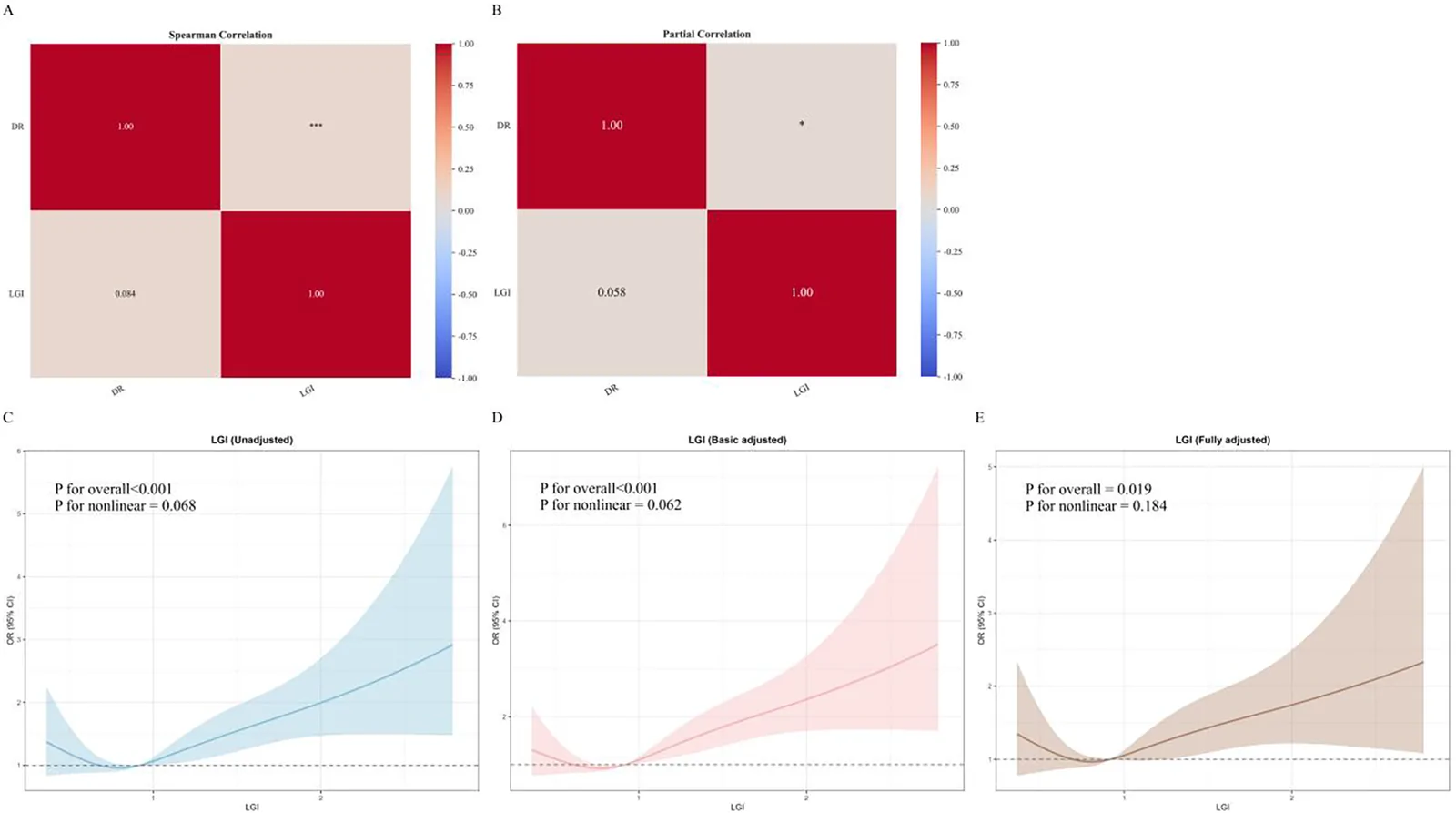
Association and dose–response relationship between LGI and prevalent DR. **(A)** Spearman correlation analysis assessing the monotonic associations between LGI and clinical variables. **(B)** Partial correlation analysis assessing the associations between LGI and DR-related variables after adjustment for age, sex, BMI, diabetes duration, insulin use, hypertension history, ALT, AST, Urea, Cr, TC, LDL-C, ALB, and HbA1c. The correlation coefficients indicate the direction and strength of the associations **(C–E) **Restricted cubic spline (RCS) analyses evaluating the dose–response relationship between LGI and prevalent DR and assessing potential departures from linearity across progressively adjusted models. **(C)** Crude model; **(D)** model adjusted for age, sex, BMI, and diabetes duration; **(E)** model further adjusted for insulin use, hypertension history, ALT, AST, Urea, Cr, TC, LDL-C, ALB, and HbA1c. The solid line represents the estimated odds ratio (OR), and the shaded area represents the corresponding 95% confidence interval (CI). *P* < 0.05, *P < 0.01, **P < 0.001. Abbreviations are listed in **Table 1**.

To comprehensively evaluate the relationship between LGI and prevalent DR, correlation analyses, multivariable logistic regression, and RCS analyses were performed. Spearman correlation analysis showed a weak but statistically significant positive correlation between LGI and prevalent DR (r = 0.084, P < 0.001; **Figure 2A**). After adjustment for potential confounders, partial correlation analysis showed that the association remained statistically significant, although the correlation remained weak (r = 0.058, P = 0.016; **Figure 2B**).

Multivariable logistic regression further confirmed a positive association between LGI and prevalent DR (**Table 2**). When LGI was analyzed as a continuous variable per one-unit increase, higher LGI was associated with a greater likelihood of prevalent DR in the unadjusted model (Model 1: OR = 1.519, 95% CI: 1.255–1.840, *P* < 0.001). The association remained significant after adjustment for age, sex, BMI, and diabetes duration (Model 2: adjusted OR = 1.735, 95% CI: 1.405–2.144, *P* < 0.001). After further adjustment for insulin use, hypertension, ALT, AST, Urea, Cr, TC, LDL-C, ALB, and HbA1c (Model 3), LGI remained independently associated with prevalent DR (adjusted OR = 1.340, 95% CI: 1.056–1.701, *P* = 0.016).

**Table 2:** Univariate and multivariate logistic analysis for the association between LGI and DR risk.

Variables	Crude model (Model1)		Basic adjusted model(Model2)		Fully adjusted model(Model3)	
	OR (95%CI)	P value	aOR (95%CI)	P value	aOR (95%CI)	P value
LGI	1.519 (1.255-1.840)	<0.001***	1.735 (1.405-2.144)	<0.001***	1.340 (1.056-1.701)	0.016*
LGI (per 1-SD increment)	1.245 (1.126-1.375)	<0.001***	1.334 (1.195-1.490)	<0.001***	1.166 (1.029-1.320)	0.016*
LGI group						
T1 (≤0.783)	1 (reference)		1 (reference)		1 (reference)	
T2(0.783-1.113)	0.970 (0.761-1.236)	0.805	1.028 (0.795-1.329)	0.834	0.971 (0.743-1.270)	0.830
T3(≥1.113)	1.506 (1.188-1.909)	<0.001***	1.830 (1.415-2.366)	<0.001***	1.378 (1.028-1.848)	0.032*
P for Trend	1.231 (1.093-1.388)	<0.001***	1.358 (1.193-1.546)	<0.001***	1.171 (1.010-1.356)	0.036*

Model 1: unadjusted, Model 2: Adjusted for age, sex, BMI and diabetes duration, Model 3: Adjusted for age, sex, BMI, diabetes duration, insulin use, Hypertension history, ALT, AST, Urea, Cr, TC, LDL-C, ALB and HbA1c. Adjusted odds ratios (aOR) were derived from multivariable logistic regression models that included all variables listed in the corresponding covariate set. Each aOR indicates the relative increase or decrease in the odds of DR per unit change in the predictor, while holding all other covariates constant. OR, odds ratio; aOR, adjusted odds ratio; CI, confidence interval; *P < 0.05, **P < 0.01, ***P < 0.001. Abbreviations as shown in **Table 1**.

When LGI was analyzed per one SD increase, the fully adjusted model also showed a significant positive association with prevalent DR (adjusted OR = 1.166, 95% CI: 1.029–1.320, *P* = 0.016), indicating the robustness of the association.

Participants were further categorized into tertiles according to LGI. Compared with the lowest tertile (T1), individuals in the highest tertile (T3, LGI ≥1.113) had a higher likelihood of prevalent DR across all three models: Model 1, OR = 1.506 (95% CI: 1.188–1.909, *P* < 0.001); Model 2, adjusted OR = 1.830 (95% CI: 1.415–2.366, *P* < 0.001); and Model 3, adjusted OR = 1.378 (95% CI: 1.028–1.848, *P* = 0.032). No significant differences were observed between the middle tertile (T2, 0.783 ≤ LGI < 1.113) and T1 in any model (*P* > 0.05). Trend analyses demonstrated that the likelihood of prevalent DR increased progressively with increasing LGI levels (all *P* for trend < 0.05).

RCS analysis was subsequently performed to examine the dose–response relationship between LGI and prevalent DR (**Figures 2C–E**). After adjustment for relevant confounders, LGI showed an overall positive association with prevalent DR, with no evidence of a nonlinear relationship (*P* for overall < 0.05; *P* for nonlinear > 0.05; **Figure 2E**). These findings were consistent with the results of the multivariable logistic regression analyses.

### 3.4 Subgroup analysis and interaction effects

To further evaluate the robustness of the association between LGI and prevalent DR, subgroup analyses were conducted according to sex, age, BMI, diabetes duration, HbA1c level, insulin use, and history of hypertension, followed by interaction tests (**Table 3**). No significant interactions were observed between LGI and any stratification variable (*P* for interaction > 0.05). Overall, the direction of the association between LGI and prevalent DR remained consistent across all subgroups, with no apparent effect modification or statistical heterogeneity, indicating that the association was relatively stable across different clinical characteristics.

**Table 3:** Subgroup analysis and interaction analysis.

Subgroup	Total	Event	LGI		
			aOR(95%CI)	P value	P for interaction
Sex					0.074
Male	1060	411	1.185 (0.893-1.605)	0.255	
Female	667	250	1.702 (1.146-2.543)	0.009**	
age					0.811
<60	1062	391	1.369 (1.002-1.881)	0.050*	
≥60	665	270	1.365 (0.961-2.010)	0.100	
BMI					0.075
<24	556	216	1.040 (0.748-1.460)	0.814	
≥24	1171	445	1.676 (1.217-2.318)	0.002**	
diabetes duration					0.846
<10	977	264	1.703 (1.229-2.367)	0.001**	
≥10	750	397	1.133 (0.828-1.594)	0.447	
HbA1c					0.067
<7	314	91	0.404 (0.117-1.176)	0.121	
≥7	1413	570	1.450 (1.132-1.874)	0.004**	
insulin use					0.507
No	819	202	1.558 (1.054-2.299)	0.026*	
Yes	908	459	1.209 (0.914-1.636)	0.201	
Hypertension history					0.616
No	956	334	1.269 (0.940-1.750)	0.134	
Yes	771	327	1.496 (1.029-2.194)	0.037*	

Adjusted for age, sex, BMI, diabetes duration, insulin use, Hypertension history, ALT, AST, Urea, Cr, TC, LDL-C, ALB and HbA1c. For subgroup analyses stratified by age, sex, BMI, diabetes duration, insulin use, or hypertension history, the corresponding stratifying variable was excluded from the adjustment set, while all other covariates remained adjusted. aOR, adjusted odds ratio; CI, confidence interval; *P < 0.05, **P < 0.01, ***P < 0.001. Abbreviations as shown in **Table 1**.

### 3.5 Development and performance evaluation of the nomogram for identifying prevalent DR

**Figure 3:**
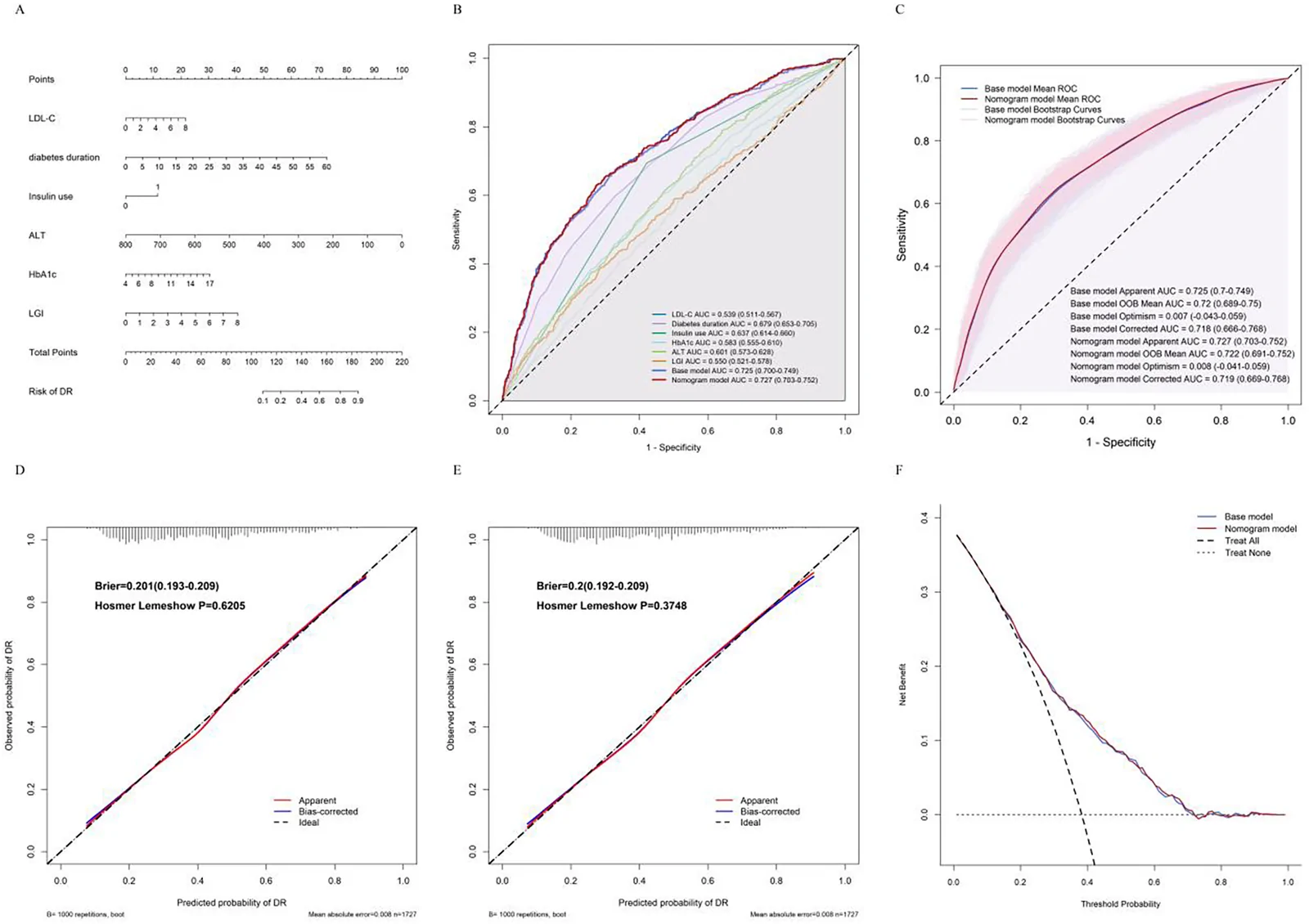
Development and internal validation of the nomogram for identifying prevalent DR. **(A)** Nomogram incorporating the selected clinical variables for estimating the probability of prevalent DR. **(B)** Receiver operating characteristic (ROC) curves showing the discriminative performance of individual predictors, the base model, and the nomogram; a higher area under the curve (AUC) indicates better discrimination. **(C)** Bootstrap-corrected ROC curves of the base model and nomogram after 1, 000 bootstrap resamples, showing their internally validated discriminative performance. **(D, E)** Calibration curves comparing the predicted probabilities with the observed proportions of prevalent DR for the base model and nomogram, respectively. Closer agreement with the reference line indicates better calibration. The black dashed line represents perfect calibration, where predicted probabilities equal the observed proportions; the solid red line represents the apparent calibration of the model; and the solid blue line represents the optimism-corrected calibration curve derived from 1, 000 bootstrap resamples. Lower Brier scores indicate better overall accuracy of probability estimates, whereas a larger Hosmer–Lemeshow P value generally indicates no evidence of poor calibration. **(F)** Decision curve analysis comparing the net benefit of the base model and nomogram across a range of threshold probabilities. Abbreviations are listed in **Table 1**.

A nomogram for identifying prevalent DR was developed based on the six variables selected by LASSO regression, including insulin use, diabetes duration, LGI, HbA1c, LDL-C, and ALT (**Figure 3A**). Comparisons of model fit, discrimination, and calibration between the base model (including insulin use, diabetes duration, HbA1c, LDL-C, and ALT) and the LGI-integrated nomogram are presented in **Supplementary Table 1** and **Figure 3**.

Compared with the base model, the nomogram showed a lower −2 log-likelihood (2032.900 vs. 2039.340) and lower Akaike information criterion (AIC) value (2044.900 vs. 2049.340), whereas the Bayesian information criterion (BIC) values were comparable (2083.080 vs. 2082.060), suggesting improved overall model fit after the inclusion of LGI (**Supplementary Table 1**).

In terms of discrimination, the apparent AUC of the nomogram was 0.727 (95% CI: 0.703–0.752), which was slightly higher than that of the base model (0.725, 95% CI: 0.700–0.749; **Figure 3B**). After 1, 000 bootstrap resamples, the optimism-corrected AUCs were 0.719 (95% CI: 0.699–0.768) for the nomogram and 0.718 (95% CI: 0.666–0.768) for the base model (**Figure 3C**). However, DeLong’s test showed that the difference in AUC between the two models was not statistically significant (ΔAUC = 0.002, *P* = 0.198; **Supplementary Table 2**).

Incremental discrimination analysis showed that adding LGI to the base model resulted in an IDI of 0.004 (95% CI: 0.001–0.007), indicating a modest but statistically significant improvement in average discrimination. The NRI was 0.114 (95% CI: 0.020–0.203), suggesting improved risk reclassification after the inclusion of LGI (**Supplementary Table 1**). However, given the small magnitude of the IDI and the lack of a statistically significant improvement in AUC, the incremental contribution of LGI to overall model performance was modest and should be interpreted cautiously.

Regarding calibration (**Figures 3D, E**), the nomogram yielded a slightly lower Brier score than the base model (0.200 [95% CI: 0.192–0.209] vs. 0.201 [95% CI: 0.193–0.209]). The Hosmer–Lemeshow goodness-of-fit test was non-significant for both the nomogram (*P* = 0.375) and the base model (*P* = 0.621), indicating good agreement between predicted and observed probabilities.

DCA demonstrated that the LGI-integrated nomogram provided a greater net benefit than the base model across a wide range of threshold probabilities (0–0.75) (**Figure 3F**), suggesting better potential clinical utility for decision-making within this range.

### 3.6 Correlations between LGI and other clinical variables

Spearman correlation analysis was further performed to investigate the relationships between LGI and other clinical variables (**Supplementary Figure 1**). LGI was positively correlated with FPG, WBC, Cr, HbA1c, TC, smoking status, ALB, ALT, AST, UA, Urea, history of hypertension, and alcohol consumption, whereas it was negatively correlated with diabetes duration, insulin use, and history of cardiovascular disease (*P* < 0.05).

## 4 Discussion

In this study, we systematically evaluated the association between the LGI and prevalent DR in patients with T2DM, and further assessed the incremental discriminative value of LGI beyond traditional clinical risk factors. The main findings showed that a higher LGI was independently associated with prevalent DR, with a positive linear dose–response relationship. This association remained generally consistent across different clinical subgroups. A nomogram incorporating insulin use, diabetes duration, HbA1c, LDL-C, ALT, and LGI, selected by LASSO regression, demonstrated acceptable discrimination and good calibration. Compared with the base model, the addition of LGI improved both the NRI and IDI, while DCA indicated greater net clinical benefit across a wide range of threshold probabilities. These findings suggest that LGI may serve as a simple composite biomarker to provide complementary information for the identification and risk stratification of prevalent DR in patients with T2DM.

LGI is derived from peripheral leukocyte count and FPG, and may simultaneously reflect abnormalities in glucose metabolism and systemic inflammatory burden. The association between LGI and DR may be related to the pathophysiological interplay between hyperglycemia and chronic inflammation.

First, the glucose component of LGI reflects the potential burden of microvascular injury associated with impaired glucose metabolism. Persistent hyperglycemia is a key pathological driver of DR and contributes to retinal microvascular damage through multiple mechanisms, including excessive activation of the polyol pathway, accumulation of advanced glycation end products, activation of protein kinase C (PKC), and enhanced oxidative stress. These processes result in retinal microvascular endothelial dysfunction, disruption of the blood–retinal barrier, pericyte loss, and capillary non-perfusion (8, 10, 30, 31). Previous studies have demonstrated that higher fasting blood glucose levels are associated with the occurrence and progression of DR. In addition, elevated HbA1c, longer diabetes duration, and increased urinary albumin-to-creatinine ratio have also been associated with DR progression (9).

Notably, in the present study, the association between LGI and prevalent DR remained statistically significant after further adjustment for HbA1c (Model 3: adjusted OR = 1.340, 95% CI: 1.056–1.701, *P* = 0.016). This finding suggests that the association between LGI and DR cannot be fully explained by long-term average glycemic control alone. Because LGI incorporates both leukocyte count and FPG, it may partially capture short-term glycemic status or other metabolic changes that are not fully reflected by HbA1c. However, as glycemic variability was not directly assessed in this study, this explanation remains speculative and requires further validation using continuous glucose monitoring and other relevant measures.

Second, the leukocyte component of LGI reflects the state of systemic low-grade inflammation. Chronic inflammation plays a central role in the development of DR, and leukocytes are critically involved in diabetes-induced vascular injury and early retinal abnormalities. In the early stages of DR, both patients with diabetes and experimental animals exhibit increased leukocyte counts and enhanced leukocyte adhesion to retinal vessels, which may contribute to retinal capillary occlusion observed in diabetes (32). Peripheral leukocytes in patients with diabetes are prone to premature activation, and their count may reflect the burden of systemic low-grade inflammation. Hyperglycemia specifically upregulates intercellular adhesion molecule-1 (ICAM-1) expression in retinal endothelial cells, promoting leukocyte adhesion and stasis. Retained leukocytes subsequently release interleukin-1β (IL-1β), prostaglandin E2 (PGE2), and superoxide anions, while activating the nuclear factor kappa B (NF-κB) pathway and increasing inducible nitric oxide synthase (iNOS) expression. These pro-inflammatory and oxidative mediators collectively impair the retinal microcirculation and accelerate the development of DR (33). Therefore, peripheral leukocyte count may, to some extent, reflect the systemic inflammatory burden associated with DR.

Previous studies have also supported the relationship between inflammation and DR from different perspectives. Several leukocyte-derived inflammatory markers, including the platelet-to-lymphocyte ratio (PLR), MLR, systemic immune-inflammation index (SII), systemic inflammation response index (SIRI), and aggregate index of systemic inflammation (AISI), have all been reported to be associated with DR (22, 34). These findings suggest that inflammatory burden may contribute to the pathogenesis of DR. Compared with single inflammatory markers, LGI integrates both leukocyte count and blood glucose levels, thereby simultaneously reflecting inflammation- and glucose metabolism-related information that may be relevant to DR. Nevertheless, it should be emphasized that, as a composite biomarker, the clinical association of LGI does not directly demonstrate a specific synergistic pathogenic mechanism between leukocytes and hyperglycemia. The underlying biological mechanisms require further investigation through basic experimental and longitudinal clinical studies.

We further observed a positive linear dose–response relationship between LGI and prevalent DR, with no evidence of a nonlinear association or threshold effect. This finding suggests that, within the observed range of this study, increasing LGI levels were associated with a progressively higher likelihood of prevalent DR, rather than becoming associated only after a specific threshold was reached. These results support the use of LGI as a continuous variable for individualized risk stratification, although its optimal clinical cutoff requires further validation in different populations and external cohorts.

In recent years, increasing attention has been given to blood-based inflammatory markers and metabolic composite indices as potential factors associated with DR. Previous studies have reported associations between inflammatory markers, such as the NLR, SII, and SIRI, and DR, whereas metabolic indices such as CHG primarily integrate glycemic and lipid-related information (9, 20–23). Compared with these indices, LGI combines peripheral leukocyte count with FPG, enabling the simultaneous incorporation of inflammation- and glucose metabolism-related information and thus providing a biologically plausible composite indicator.

Previous studies have explored the clinical significance of LGI in various disease settings (14–19). For example, in patients with ST-segment elevation myocardial infarction, LGI was associated with in-hospital mortality (17). In patients with COVID-19, LGI was identified as an independent risk factor for severe disease (OR = 1.727, 95% CI: 1.026–3.048; AUC = 0.749), with substantially improved predictive performance in the subgroup with diabetes (AUC = 0.915), indicating that LGI is a strong predictor of disease severity in patients with diabetes (15). In patients with diabetic ketoacidosis (DKA), elevated LGI was associated with both DKA severity (OR = 1.87, *P* = 0.016) and diabetic microvascular complications (OR = 2.16, *P* = 0.010) *(*18). Collectively, these findings suggest that LGI may serve as a systemic inflammation–metabolism risk marker. Building upon this evidence, the present study further identified an independent association between LGI and prevalent DR in patients with T2DM and established a corresponding nomogram for identifying prevalent DR, thereby extending the potential application of LGI in diabetic microvascular complications. However, as this was a cross-sectional study, the findings should be interpreted as a statistical association between LGI and prevalent DR rather than evidence that LGI is a causal or independent pathogenic factor for DR. Prospective studies are needed to determine whether LGI can predict the future onset or progression of DR.

Using LASSO regression, we selected six variables, including insulin use, diabetes duration, HbA1c, LGI, LDL-C, and ALT, to construct a nomogram for identifying prevalent DR. The model achieved an apparent AUC of 0.727, and the optimism-corrected AUC after 1, 000 bootstrap resamples was 0.719, indicating acceptable discrimination. The model also demonstrated good calibration, with a Brier score of 0.200 and no evidence of poor fit according to the Hosmer–Lemeshow test. Compared with the base model, which included insulin use, diabetes duration, HbA1c, LDL-C, and ALT, the addition of LGI increased the AUC by only 0.002, and the difference was not statistically significant according to the DeLong test (P = 0.198). These findings indicate that the improvement in overall discrimination provided by LGI was limited. Therefore, the results should not be interpreted as showing that LGI significantly improved the overall AUC of the model. In contrast, both NRI and IDI analyses demonstrated statistically significant improvements in risk reclassification and average discrimination after the inclusion of LGI, suggesting that LGI may provide complementary information beyond traditional clinical risk factors. AUC, NRI, and IDI evaluate different aspects of model performance. AUC primarily reflects the overall ranking and discriminative ability of a model, whereas NRI and IDI assess the incremental value of a new marker from the perspectives of risk reclassification and separation of predicted probabilities. Therefore, differences among these performance metrics are not necessarily contradictory. Nevertheless, the clinical implications of NRI and IDI should also be interpreted with caution, as they depend on the study population, model specification, and intended clinical application. Accordingly, our findings are more appropriately interpreted as demonstrating that LGI may provide complementary information beyond traditional clinical factors, although its incremental contribution to overall discrimination was limited. DCA further showed that the nomogram incorporating LGI achieved greater net benefit than the base model across a range of threshold probabilities. This finding suggests that, if used to assist in identifying patients requiring further fundus evaluation, the incorporation of LGI may have potential clinical utility. However, because prospective implementation studies have not yet been performed, whether the model can improve screening efficiency, reduce healthcare costs, or improve patient outcomes remains to be determined.

The present findings also have potential clinical implications. Current DR screening relies heavily on retinal imaging, which may be difficult to implement in resource-limited settings. Because LGI can be readily calculated from routine complete blood count and FPG measurements without additional cost or specialized equipment, it may serve as an initial risk stratification tool for DR. Patients with elevated LGI could be prioritized for retinal imaging, thereby improving the efficiency of limited ophthalmic resources, whereas patients with lower LGI levels might be considered for appropriately extended screening intervals, potentially reducing the burden on healthcare systems. Such a biomarker-based stratified screening strategy may be more cost-effective than the current uniform annual screening approach. In addition, as a dynamic laboratory indicator, longitudinal changes in LGI may provide more clinically relevant information than a single measurement. Future studies should investigate the association of temporal changes and cumulative exposure to LGI with the incidence and progression of DR, and determine whether repeated LGI measurements can improve dynamic risk assessment.

This study has several strengths. First, we focused on an easily obtainable composite blood biomarker and systematically evaluated the association and dose–response relationship between LGI and prevalent DR in patients with T2DM, providing new clinical evidence supporting the combined use of inflammation- and glucose metabolism-related information for DR risk stratification. Second, multiple complementary analytical approaches, including multivariable logistic regression, RCS analysis, subgroup analysis, and interaction testing, were used to comprehensively evaluate the association, dose–response relationship, and potential effect modification. Variables selected by LASSO regression were used to construct a nomogram, and model performance was comprehensively assessed using bootstrap internal validation, calibration analysis, NRI, IDI, and DCA. Finally, because LGI is derived from routine laboratory parameters and does not require specialized testing, it is readily accessible and has promising translational potential, particularly in resource-limited healthcare settings.

Several limitations should also be acknowledged. First, this was a single-center cross-sectional study of hospitalized patients with T2DM. Therefore, causal relationships between LGI and DR cannot be established. In addition, because participants were recruited from a hospital setting rather than from the general population, our findings cannot be directly extrapolated to the broader T2DM population. Hospital-based recruitment may have introduced selection bias, particularly hospitalization-related selection bias and referral bias, as patients admitted to or referred to a tertiary hospital may differ systematically from community-dwelling or non-hospitalized patients in terms of disease severity, comorbidity burden, metabolic control, and treatment characteristics. Furthermore, the inclusion of participants with complete data for the variables required for analysis may have introduced additional selection bias, as patients with complete data may differ systematically from those with incomplete or missing data in their clinical characteristics. These selection mechanisms may have limited the external validity of our findings and may have influenced the observed association between LGI and prevalent DR. Therefore, the present findings should be interpreted within the context of the study population, and future multicenter studies involving community-based and outpatient populations are warranted to determine whether the observed association can be generalized to the broader T2DM population. Second, LGI was calculated from a single measurement of total leukocyte count and FPG and may therefore be influenced by short-term glycemic fluctuations and other transient physiological factors. Moreover, total leukocyte count is a relatively nonspecific marker of systemic inflammation and does not capture the potentially distinct contributions of individual leukocyte subtypes. Differential leukocyte counts, including neutrophils, lymphocytes, and monocytes, were not incorporated into the present analysis. Therefore, whether specific leukocyte subpopulations provide additional information beyond total leukocyte count or improve the identification of prevalent DR remains unclear. Future studies should incorporate differential leukocyte profiles and repeated measurements of LGI to determine whether specific leukocyte subtypes or longitudinal changes in LGI provide additional information regarding the incidence and progression of DR. Third, although multiple important demographic, clinical, and laboratory variables were considered in the multivariable analyses, residual confounding cannot be completely excluded. Total leukocyte count may be influenced by immune status, subclinical inflammatory conditions, lifestyle characteristics, and treatment-related factors. Several potentially relevant factors, including dietary patterns, physical activity, detailed changes in diabetes treatment, inflammatory biomarkers, and other diabetes-related complications, were not fully accounted for in the present study. Therefore, the observed association between LGI and prevalent DR should not be interpreted as evidence of a causal relationship, and future studies incorporating more comprehensive inflammatory, lifestyle-related, and treatment-related information are warranted. Fourth, DR severity was not further classified into non-proliferative DR and proliferative DR, precluding assessment of whether the association between LGI and DR differs across disease stages. Future studies with larger sample sizes should further investigate the relationship between LGI and DR severity and progression. Finally, only internal validation was performed, and no independent external validation cohort was available. Therefore, although the nomogram demonstrated acceptable discrimination and good calibration, its generalizability across different regions, healthcare settings, and populations remains to be established. Future multicenter prospective studies with external validation are needed to evaluate the robustness and clinical utility of LGI and the nomogram for the identification and risk stratification of prevalent DR.

## 5 Conclusion

In patients with T2DM, a higher LGI was independently associated with prevalent DR, with a stable positive linear dose–response relationship. This association remained generally consistent across different clinical subgroups, suggesting that LGI may reflect the combined burden of inflammation and impaired glucose metabolism related to DR. A nomogram incorporating diabetes duration, insulin use, LDL-C, ALT, HbA1c, and LGI demonstrated acceptable discrimination and good calibration. Although the addition of LGI resulted in only a modest and statistically non-significant improvement in the overall AUC, NRI and IDI analyses indicated that LGI provided incremental discriminative value beyond traditional clinical risk factors, and DCA suggested additional net benefit across a range of clinically relevant threshold probabilities. Therefore, as a simple composite index derived from routine laboratory measurements, LGI may provide complementary information for the identification and individualized risk stratification of prevalent DR in patients with T2DM. However, given the single-center cross-sectional design and the lack of external validation, whether LGI can predict the future onset or progression of DR and its clinical utility in routine screening require further confirmation in multicenter prospective studies.

## Data Availability

The original contributions presented in the study are included in the article/**Supplementary Material**. Further inquiries can be directed to the corresponding author.

## Supplementary material

Supplementary Table 1 Comparison of model performance between the base model and the full nomogram model for identifying prevalent DR Note: −2LL, −2 times the log-likelihood, reflects model fit, with lower values generally indicating better fit. AIC, Akaike information criterion, evaluates model fit while penalizing model complexity, with lower values indicating a better balance between goodness of fit and model complexity. BIC, Bayesian information criterion, similarly evaluates model fit while imposing a stronger penalty for model complexity, with lower values generally indicating a preferable model. AUC, area under the receiver operating characteristic curve, reflects the model’s ability to discriminate between patients with and without prevalent DR. Bootstrap-corrected AUCs were obtained using 1, 000 bootstrap resamples for internal validation. NRI, net reclassification improvement, quantifies the improvement in risk reclassification after adding LGI to the base model. IDI, integrated discrimination improvement, quantifies the improvement in the average separation of predicted probabilities between patients with and without prevalent DR after adding LGI to the base model. Brier score measures the overall accuracy of probability estimates, with lower values indicating better overall performance. The Hosmer–Lemeshow test assesses the agreement between predicted and observed probabilities, with a larger P value generally indicating no evidence of poor calibration. NRI and IDI were calculated for the nested comparison between the base model and the nomogram model with additional LGI. CI, confidence interval. Abbreviations are defined in **Table 1**.

Supplementary Table 2 Comparison of the AUCs between the base model and the nomogram model using the DeLong test The DeLong test was used to compare the AUCs of the base model and the nomogram model. A positive AUC difference indicates a higher AUC for the nomogram model than for the base model. A *P* value < 0.05 was considered statistically significant. AUC, area under the receiver operating characteristic curve; vs, versus; Abbreviations are listed in **Table 1**.

## References

[1] Genitsaridi I Salpea P Salim A Sajjadi SF Tomic D James S . 11th edition of the IDF Diabetes Atlas: global, regional, and national diabetes prevalence estimates for 2024 and projections for 2050. Lancet Diabetes Endocrinol. (2026) 14(2):149–56. doi: 10.1016/s2213-8587(25)00299-2 41412135

[2] Cao J Liu F Kong H Lai C Du Z Soh ZD . Integrated intraocular-plasma proteomics reveals conserved biomarkers for diabetic retinopathy progression: a multi-fluid biopsy study. Diabetologia. (2026) 69(7):1823–38. doi: 10.1007/s00125-026-06708-3 41817688

[3] Cui X Wen D Xiao J Li X . The causal relationship and association between biomarkers, dietary intake, and diabetic retinopathy: insights from Mendelian randomization and cross-sectional study. Diabetes Metab J. (2025) 49(5):1087–105. doi: 10.4093/dmj.2024.0731 40160026 PMC12436034

[4] Invernizzi A Chhablani J Viola F Gabrielle PH Zarranz-Ventura J Staurenghi G . Diabetic retinopathy in the pediatric population: pathophysiology, screening, current and future treatments. Pharmacol Res. (2023) 188:106670. doi: 10.1016/j.phrs.2023.106670 36681366

[5] Zhao X Gu X Teng D Sun X Wei Q Wang B . Ultra-wide-field fundus photography and AI-based screening and referral for multiple ocular fundus diseases. Cell Rep Med. (2025) 6(6):102187. doi: 10.1016/j.xcrm.2025.102187 40499544 PMC12208325

[6] Lin KY Hsih WH Lin YB Wen CY Chang TJ . Update in the epidemiology, risk factors, screening, and treatment of diabetic retinopathy. J Diabetes Investig. (2021) 12(8):1322–5. doi: 10.1111/jdi.13480 33316144 PMC8354492

[7] Wang S Jia B Niu S Chen S . Relationship between the hemoglobin, albumin, lymphocyte count, platelet count (HALP) score and type 2 diabetes retinopathy. Diabetes Metab Syndr Obes. (2024) 17:2693–706. doi: 10.2147/dmso.S467799 39007156 PMC11246656

[8] Zhu H Li B Huang T Wang B Li S Yu K . Update in the molecular mechanism and biomarkers of diabetic retinopathy. Biochim Biophys Acta Mol Basis Dis. (2025) 1871(5):167758. doi: 10.1016/j.bbadis.2025.167758 40048937

[9] Mersha GA Molla MD Chakraborty R Kaidonis G Lake S Craig J . Incidence, progression and determinants of diabetic retinopathy in type 2 diabetes in Australasia: a systematic review and meta-analysis. Clin Exp Ophthalmol. (2026) 54(4):460–81. doi: 10.1111/ceo.70095 41787767

[10] Guo C Sodhi A . Molecular stress and neurovascular injury in the diabetic retina. J Clin Invest. (2026) 36(5). doi: 10.1172/jci200945 41766669 PMC12948430

[11] Wang Z Guo H Teng X . Diabetic retinopathy and mortality in adults with diabetes: causal mediation analysis of the role of HbA1c. Diabetes Res Clin Pract. (2025) 229:112936. doi: 10.1016/j.diabres.2025.112936 41075986

[12] Begum T Rahman A Nomani D Mamun A Adams A Islam S . Diagnostic accuracy of detecting diabetic retinopathy by using digital fundus photographs in the peripheral health facilities of Bangladesh: validation study. JMIR Public Health Surveill. (2021) 7(3):e23538. doi: 10.2196/23538 33411671 PMC7988391

[13] Brinchmann-Hansen O Dahl-Jørgensen K Sandvik L Hanssen KF . Blood glucose concentrations and progression of diabetic retinopathy: the seven year results of the Oslo study. Bmj. (1992) 304(6818):19–22. doi: 10.1136/bmj.304.6818.19 1734985 PMC1880908

[14] Mureşan AV Florea E Arbănaşi EM Bartus R Arbănaşi EM Ion AP . Elevated leukocyte glucose index is associated with long-term arteriovenous fistula failure in dialysis patients. J Clin Med. (2024) 13(7). doi: 10.3390/jcm13072037 38610802 PMC11012331

[15] Ramos-Hernández WM Soto LF Del Rosario-Trinidad M Farfan-Morales CN De Jesús-González LA Martínez-Mier G . Leukocyte glucose index as a novel biomarker for COVID-19 severity. Sci Rep. (2022) 12(1):14956. doi: 10.1038/s41598-022-18786-5 36056114 PMC9438363

[16] Omar MB Eynel E Nasif S Toprak K Taşcanov MB . Prognostic capacity of the leuko-glycemic index in cardiac syndrome Y. Sci Prog. (2025) 108(3):368504251356562. doi: 10.1177/00368504251356562 40685606 PMC12277937

[17] Aygun A Saribas MS Tomakin M Koksal A Akkaya F Caltekin I . Assessment of inflammatory and glycemic markers for in-hospital mortality in STEMI patients. Ir J Med Sci. (2026) 195(2):687–94. doi: 10.1007/s11845-025-04272-0 41533267

[18] Coşarcă MC Tilinca RM Lazăr NA Şincaru SV Bandici BC Caraşca C . Elevated leukocyte glucose index (LGI) is associated with diabetic ketoacidosis (DKA) severity and presence of microvascular complications. Med (Kaunas). (2025) 61(5). doi: 10.3390/medicina61050898 40428856 PMC12113475

[19] Ye X Chen S Guo L Ma X Wu L Li Y . Association of leuko-glycemic index with mortality in ICU patients with acute kidney injury: a retrospective multicenter cohort study. PloS One. (2026) 21(6):e0350811. doi: 10.1371/journal.pone.0350811 42241422 PMC13235893

[20] Dascalu AM Serban D Tanasescu D Vancea G Cristea BM Stana D . The value of white cell inflammatory biomarkers as potential predictors for diabetic retinopathy in type 2 diabetes mellitus (T2DM). Biomedicines. (2023) 11(8). doi: 10.3390/biomedicines11082106 37626602 PMC10452280

[21] Deng R Zhu S Fan B Chen X Lv H Dai Y . Exploring the correlations between six serological inflammatory markers and different stages of type 2 diabetic retinopathy. Sci Rep. (2025) 15(1):1567. doi: 10.1038/s41598-025-85164-2 39794420 PMC11723939

[22] Bulu A Keser S . The relationship between pan-immune inflammation value and different stages of diabetic retinopathy in patients with type 2 diabetes mellitus: a prospective cross-sectional study. BMC Endocr Disord. (2025) 25(1):184. doi: 10.1186/s12902-025-02007-x 40702514 PMC12285020

[23] Çatak M Konuk Ş G Hepsen S . The cholesterol-HDL-glucose (CHG) index and traditional adiposity markers in predicting diabetic retinopathy and nephropathy. J Diabetes Investig. (2025) 16(8):1487–94. doi: 10.1111/jdi.70086 40434226 PMC12315246

[24] Cai XT Ji LW Liu SS Wang MR Heizhati M Li NF . Derivation and validation of a prediction model for predicting the 5-year incidence of type 2 diabetes in non-obese adults: a population-based cohort study. Diabetes Metab Syndr Obes. (2021) 14:2087–101. doi: 10.2147/dmso.S304994 34007195 PMC8123981

[25] Mugo P Jeilan M Msunza M Orwa J Ngunga M . Incidence of bleeding and performance of the PRECISE-DAPT score in predicting bleeding in patients on dual antiplatelet therapy after treatment for acute coronary syndrome in Kenya. BMC Cardiovasc Disord. (2025) 25(1):137. doi: 10.1186/s12872-024-04434-5 40016650 PMC11869397

[26] Yu P Kan R Meng X Wang Z Xiang Y Mao B . A nomogram for predicting the risk of CKD based on cardiometabolic risk factors. Int J Gen Med. (2023) 16:4143–54. doi: 10.2147/ijgm.S425122 37720178 PMC10503556

[27] Perezalonso-Espinosa J Ramírez-García D Díaz-Sánchez JP Carrillo-Herrera KB Cabrera-Quintana LA Dávila-López G . External validation and recalibration of cardiovascular risk scores for prediction of 10-year risk of fatal cardiovascular disease: a prospective, observational, population-based cohort analysis of adults in Mexico City. Lancet Reg Health Am. (2026) 56:101403. doi: 10.1016/j.lana.2026.101403 41732703 PMC12925590

[28] Zhang M Lei N Zhang XL Xu Y Chen HF Fu LZ . Developing and validating a prognostic prediction model for patients with chronic kidney disease stages 3-5 based on disease conditions and intervention methods: a retrospective cohort study in China. BMJ Open. (2022) 12(5):e054989. doi: 10.1136/bmjopen-2021-054989 35636798 PMC9153056

[29] Song R Ni H Huang J Yang C Qin S Wei H . Prognostic value of inflammation-immunity-nutrition score and inflammatory burden index for hepatocellular carcinoma patients after hepatectomy. J Inflammation Res. (2022) 15:6463–79. doi: 10.2147/jir.S386407 36467989 PMC9717599

[30] Toma C Ferdeghini D Ola Pour MM Venkatesan S De Cillà S Grossini E . Oxidative stress in diabetic retinopathy: pathogenic mechanisms, biomarkers and clinical implications. Antioxidants (Basel). (2026) 15(4). doi: 10.3390/antiox15040425 42072067 PMC13113219

[31] Sheng X Zhang C Zhao J Xu J Zhang P Ding Q . Microvascular destabilization and intricated network of the cytokines in diabetic retinopathy: from the perspective of cellular and molecular components. Cell Biosci. (2024) 14(1):85. doi: 10.1186/s13578-024-01269-7 38937783 PMC11212265

[32] Saadane A Veenstra AA Minns MS Tang J Du Y Abubakr Elghazali F . CCR2-positive monocytes contribute to the pathogenesis of early diabetic retinopathy in mice. Diabetologia. (2023) 66(3):590–602. doi: 10.1007/s00125-022-05860-w 36698021 PMC9892100

[33] Lessieur EM Liu H Saadane A Du Y Kiser J Kern TS . ICAM-1 on the luminal surface of endothelial cells is induced to a greater extent in mouse retina than in other tissues in diabetes. Diabetologia. (2022) 65(10):1734–44. doi: 10.1007/s00125-022-05719-0 35852587 PMC9481679

[34] Gong Y Wang L Li Q Wang M Luan R Gao F . Evaluating neutrophil-lymphocyte ratio, systemic immune-inflammation index, and systemic inflammation response index for diagnosing and predicting progression in diabetic retinopathy: a cross-sectional and longitudinal study. BMC Ophthalmol. (2025) 25(1):398. doi: 10.1186/s12886-025-04222-5 40629269 PMC12239467

