# Supplementary material for: The leukocyte glucose index: a novel inflammatory-glucose biomarker for prevalent diabetic retinopathy and its supplementary predictive capacity in individuals with type 2 diabetes mellitus
Source: Front Endocrinol (Lausanne). 2026 Sep 7;17:1959346. doi: 10.3389/fendo.2026.1959346 (PMC13590353; doi:10.3389/fendo.2026.1959346)
Supplement: Supplementary file 1 [file Supplementaryfile1.docx]

**Supplementary Figure 1. Spearman correlation analysis between LGI and clinical variables.**

Spearman correlation was used to assess the monotonic association between LGI and each clinical variable. The correlation coefficients indicate the direction and strength of the associations. *P < 0.05, **P < 0.01, ***P < 0.001. Abbreviations are listed in Table 1.
