# Supplementary material for: The leukocyte glucose index: a novel inflammatory-glucose biomarker for prevalent diabetic retinopathy and its supplementary predictive capacity in individuals with type 2 diabetes mellitus
Source: Front Endocrinol (Lausanne). 2026 Sep 7;17:1959346. doi: 10.3389/fendo.2026.1959346 (PMC13590353; doi:10.3389/fendo.2026.1959346)
Supplement: Supplementary file 2 [file Table1.docx]

**Supplementary Table 1. Comparison of model performance between the base model and the full nomogram model for identifying prevalent DR**

| Metric | Base Model | Nomogram Model |
| --- | --- | --- |
| −2LL | 2039.340 | 2032.900 |
| AIC | 2049.340 | 2044.900 |
| BIC | 2082.060 | 2083.080 |
| Apparent_AUC (95% CI) | 0.725 (0.700—0.749) | 0.727 (0.703—0.752) |
| Corrected_AUC (95% CI) | 0.718 (0.666—0.768) | 0.719 (0.669—0.768) |
| Brier (95% CI) | 0.201 (0.193—0.209) | 0.200 (0.192—0.209) |
| Hosmer–Lemeshow P value | 0.621 | 0.375 |
| Total NRI (95% CI) | — | 0.114 (0.020—0.203) |
| Total IDI (95% CI) | — | 0.004 (0.001—0.007) |

Note: −2LL, −2 times the log-likelihood, reflects model fit, with lower values generally indicating better fit. AIC, Akaike information criterion, evaluates model fit while penalizing model complexity, with lower values indicating a better balance between goodness of fit and model complexity. BIC, Bayesian information criterion, similarly evaluates model fit while imposing a stronger penalty for model complexity, with lower values generally indicating a preferable model. AUC, area under the receiver operating characteristic curve, reflects the model’s ability to discriminate between patients with and without prevalent DR. Bootstrap-corrected AUCs were obtained using 1,000 bootstrap resamples for internal validation. NRI, net reclassification improvement, quantifies the improvement in risk reclassification after adding LGI to the base model. IDI, integrated discrimination improvement, quantifies the improvement in the average separation of predicted probabilities between patients with and without prevalent DR after adding LGI to the base model. Brier score measures the overall accuracy of probability estimates, with lower values indicating better overall performance. The Hosmer–Lemeshow test assesses the agreement between predicted and observed probabilities, with a larger P value generally indicating no evidence of poor calibration. NRI and IDI were calculated for the nested comparison between the base model and the nomogram model with additional LGI. CI, confidence interval. Abbreviations are defined in Table 1.
