# Supplementary material for: The leukocyte glucose index: a novel inflammatory-glucose biomarker for prevalent diabetic retinopathy and its supplementary predictive capacity in individuals with type 2 diabetes mellitus
Source: Front Endocrinol (Lausanne). 2026 Sep 7;17:1959346. doi: 10.3389/fendo.2026.1959346 (PMC13590353; doi:10.3389/fendo.2026.1959346)
Supplement: Supplementary file 3 [file Table2.docx]

**Supplementary Table 2. Comparison of the AUCs between the base model and the nomogram model using the DeLong test**

| Comparison | Base Model AUC | Nomogram Model AUC | AUC Difference | P value |
| --- | --- | --- | --- | --- |
| Base model vs Nomogram model | 0.725 | 0.727 | 0.002 | 0.198 |

Note: The DeLong test was used to compare the AUCs of the base model and the nomogram model. A positive AUC difference indicates a higher AUC for the nomogram model than for the base model. A *P* value < 0.05 was considered statistically significant. AUC, area under the receiver operating characteristic curve; vs, versus; Abbreviations are listed in Table 1.
